# Unveiling the potential of apigenin and kaempferol against colon cancer: an integrated network pharmacology and docking approach

**DOI:** 10.3389/fbinf.2026.1702572

**Published:** 2026-02-13

**Authors:** Anushya Selvakumar, Perpetual Ansel Chandran, Sai Shraddha, Loganathan Chandramani Priya Dharshini, Sarath Perumal, Ramanathan Karuppasamy, Abul Kalam Azad Mandal

**Affiliations:** Department of Biotechnology, School of Bio Sciences and Technology, Vellore Institute of Technology, Vellore, Tamil Nadu, India

**Keywords:** apigenin, Camellia sinensis, colon cancer, kaempferol, molecular docking, molecular dynamics simulation, network pharmacology

## Abstract

**Background:**

Colon cancer is one of the prevalent and deadly malignancies, requiring advanced treatment strategies.

**Methods:**

IMPPAT database, drug-likeliness, bioavailability scores, and Lipinski/Ghosh rules were utilized to screen the phytochemicals. STITCH, SwissTargetPrediction, CTD, and GeneCards were utilized for target gene retrieval (Apigenin and Kaempferol). From GeneCards, OMIM, and the NCBI Ensembl database, colon cancer-related genes were collected. The PPI network was built from the overlapping genes using STRING and Cytoscape. 10 hub genes were screened using the MCC algorithm and subjected to functional enrichment and mutation frequency analysis. Genes with high mutation frequency were selected for molecular docking and MDS.

**Results:**

A total of 292 overlapping targets between the two compounds and colon cancer-related genes were identified. The PPI network resulted in ten hub genes (AKT1, IL6, JUN, NFKB1, STAT3, TNF, BCL2, IL1B, HIF1A, and TGFB1). These were significantly enriched in key oncogenic pathways. Mutation frequency analysis revealed recurrent alterations in AKT1, NFKB1, and HIF1A. Docking studies showed strong binding of Apigenin and Kaempferol with AKT1, exhibiting binding energies of −9.4 and −9.2 kcal/mol, respectively. To further assess the binding stability of the apigenin–AKT1 complex, a 100 ns MDS was performed, which confirmed the structural stability.

**Conclusion:**

Apigenin and kaempferol showed potential as dual-targeting agents for colon cancer therapy. Cell culture and animal model studies in future are warranted to substantiate the mechanistic roles in tumor suppression.

## Graphical Abstract

**Figure:**
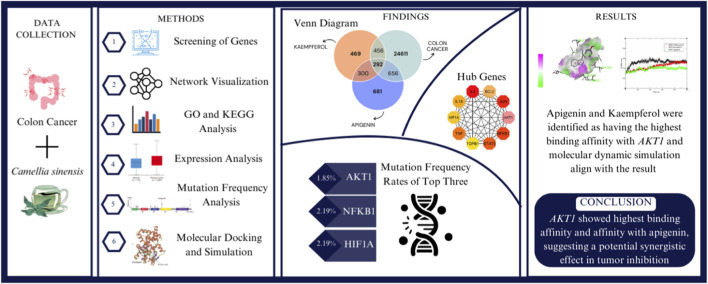
Network pharmacology identified the top overlapping genes between apigenin and kaempferol in colon cancer. Docking and MDS analysis suggested that targeting *AKT1* will be an effective therapeutic approach.

## 1 Introduction

Colon cancer ranks top, closely after lung and bronchus cancer (National Cancer Institute, 2024). Despite significant advancements in science and technology, colon cancer continues to pose a substantial public health challenge. As of 2022, the global burden of colon cancer is approximately 19,26,425 new cases and 9,04,019 deaths, ranking third among males and females globally (Sathishkumar et al., 2023; Ferlay et al., 2024). However, in India, colon cancer does not rank among the top five cancers. Though chemotherapy and surgical resection are employed in the early stages of cancer, their efficacy is impeded by various constraints. Chemotherapy induces off-target toxicity, DNA damage, and triggers stress-related pathways, resulting in therapeutic resistance (Chakrabarti et al., 2020; Islam et al., 2020).

In these circumstances, an exemplar is combination therapy, which overcomes monotherapy resistance and targets multiple pathways related to tumorigenesis. Natural phytochemicals, specifically flavonoids, have gained attention due to their multitargeted approach, low toxicity profiles, and modulation of key molecular processes in cancer progression. Among these, Apigenin and Kaempferol, two major flavonoids abundant in *Camellia sinensis* (green tea), are well-known for its antioxidant, anti-inflammatory, and anticancer properties. However, their potential as dual-targeting agents for colon cancer remains unexplored. Green tea with higher bioactives has been utilized for decades in traditional medicine across China, India, and African and Asian regions (Trisha et al., 2022; Valavanidis, 2019). Rich storage of polyphenolic compounds with anticancer activity, remarkably in apoptotic induction, angiogenesis inhibition, and immune response modulation (Esmeeta et al., 2022; Rafiq et al., 2023). Also, known for its effect on cardiovascular diseases, offering an increase in resistance of the low-density lipoprotein (LDL) present in the plasma towards oxidation, reducing the chances of atherogenesis (Namita et al., 2012).

Network pharmacology is a current method used to construct protein–gene networks to uncover the different mechanisms that can be targeted for therapeutic response. This approach unravels the putative targets through computational and experimental validations (Noor et al., 2022). This *in silico* method offers several advantages, including easy model building to study the effects of drugs and flexible network structures that facilitate a deeper understanding of signaling pathways, leading to a higher chance of therapeutic success (Zhang et al., 2013).

The present study leverages network pharmacology integrated with docking and simulations to decode multi-target interactions of Apigenin and Kaempferol in colon cancer. By employing a systems biology approach, we identified key overlapping and unique targets of these flavonoids. Hub genes were further prioritized based on expression profiles, survival analysis, and mutation frequency. Subsequent molecular docking and dynamic simulation studies were conducted to evaluate the binding and structural stability with targets, benchmarking them against a standard colon cancer drug. The experimental flowchart is portrayed inFigure 1.

**FIGURE 1:**
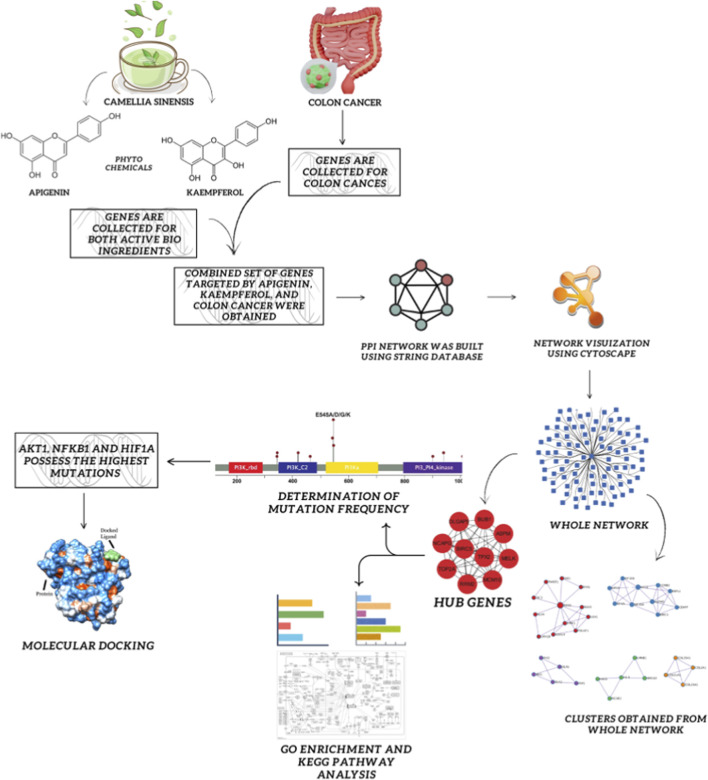
Systematic representation depicting the study’s comprehensive flow.

## 2 Materials and methods

### 2.1 Selection of phytochemicals from Camellia sinensis

Phytochemicals of *C. sinensis* (tea plant) were retrieved from the Indian Medicinal Plants, Phytochemistry and Therapeutics 2.0 (IMPPAT 2.0) database (https://cb.imsc.res.in/imppat/), which provides data on bioavailability, drug-likeness, and adherence to the Ghose rule and Lipinski’s rule of 5 (Mohanraj et al., 2018). The active phytochemicals in the leaf were selected based on their availability and further screened for Ghose and Lipinski compliance, followed by drug-likeliness score (DL) and bioavailability score (OB), with a threshold of ≥0.55 (Vivek-Ananth et al., 2023). Further anticancer-relevant candidates were refined based on flavonoid characteristics, and a topological polar surface area (TPSA) > 60, as moderately polar compounds typically fall within this range, exhibiting solubility and permeability balance.

### 2.2 Screening of putative genes for selected phytochemicals and colon cancer

Target genes for apigenin and kaempferol were retrieved from STITCH (http://stitch.embl.de/), SwissTargetPrediction (http://www.swisstargetprediction.ch/), Comparative Toxicogenomics Database (https://ctdbase.org/), and GeneCards (https://www.genecards.org/). Duplicates were removed to get refined datasets. Colon cancer-associated genes were collected from GeneCards (https://www.genecards.org/), Online Mendelian Inheritance in Man (OMIM) (https://www.omim.org/), Ensembl (https://asia.ensembl.org/index.html), and National Center for Biotechnology Information (NCBI) (https://www.ncbi.nlm.nih.gov/gene). After duplicate removal, overlapping targets were identified for apigenin-colon cancer, kaempferol-colon cancer, and apigenin-kaempferol.

### 2.3 PPI network construction

PPI network was constructed using STRING database (https://string-db.org/), set at a minimum confidence score (0.4) and organism “*Homo sapiens*”. Default parameters were applied to obtain the protesin interaction network (PIN).

### 2.4 Identification of hub genes and core regulatory modules

The PIN was imported into Cytoscape 3.10.2 (https://cytoscape.org/) for visualization and analysis of the network (Shannon et al., 2003). Compound-target networks were visualized using continuous mapping. Top 10 hub genes were pointed out through ‘CytoHubba’ with Maximal Clique Centrality (MCC) method (https://apps.cytoscape.org/apps/cytohubba). Node size was scaled by the ‘in-degree’ score. Core regulatory modules were identified using MCODE’s (https://apps.cytoscape.org/apps/mcode) default parameters.

### 2.5 Functional enrichment analysis

Gene Ontology (biological process, molecular function, and cellular component), and KEGG enrichment analysis were performed using ShinyGO 0.80 (http://bioinformatics.sdstate.edu/go/) (Ge et al., 2020; Luo and Brouwer, 2013). Statistically significant terms were filtered (FDR cutoff- 0.05), and the topmost 10 pathways were reported.

### 2.6 Mutation frequency analysis of hub genes in COAD

Mutation frequency analysis was assessed in the colon adenocarcinoma (COAD) dataset of cBioPortal (https://www.cbioportal.org/) (De Bruijn et al., 2023), encompassing 10967 from a predefined patient list, and data regarding deep deletions, multiple alterations, amplification, structure variants, and mutations were generated. The top three most frequently mutated genes were selected for further analysis, as mutation frequency offers a stable genomic indicator in contrast to expression- or survival-based metrics.

### 2.7 Molecular docking

Three key genes *AKT1*, *NFKB1*, and *HIF1A* were docked with apigenin (PubChem ID: 5280443) and kaempferol (PubChem ID: 5280863). 3D conformer structures of Apigenin and Kaempferol were obtained from PubChem (https://pubchem.ncbi.nlm.nih.gov/). The PDB 3D structures for the targeted genes were downloaded from RCSB PDB (https://www.rcsb.org/) using PDB IDs 6HHF, 8TQD, and 1LQB for *AKT1*, *NFKB1*, and *HIF1A*, respectively. As the structures were available as co-crystallized complexes, all bound inhibitors, ligands, and non-essential heteroatoms were removed prior to docking, and only the required protein chains were retained.

As a reference control, 5-Fluorouracil (PubChem ID: 3385) was included, as it is a first-line chemotherapeutic agent widely used in the clinical management of colon cancer and serves as a standard benchmark for comparative *in silico* evaluation of anticancer compounds. The protein structures were prepared in Auto Dock tools 1.5.7 (removal of water molecules, adding polar hydrogens, and calculating Kollman charges) (Forli et al., 2016). Blind docking was performed using the PyRx 0.8 Virtual Screening tool, and interactions were visualized in BIOVIA Discovery Studio Visualizer (Dallakyan and Olson, 2015; Dassault Systemes BIOVIA, 2021).

### 2.8 Estimation of binding free energy

Binding free-energy calculations were performed using the Uni-GBSA workflow to obtain a refined estimation of protein–ligand interaction energetics. The binding free energy was evaluated using the following equation:

$$\Delta G_{bind}=\Delta G_{complex}-\Delta G_{protein}-\Delta G_{ligand}$$

where ΔG bind is the ligand-binding energy, ΔG complex is the energy of the complex, ΔG protein is the energy of the receptor without the ligand, and ΔG ligand is the energy of the unbound ligand (Wang et al., 2006).

### 2.9 Molecular dynamics simulation analysis

Molecular dynamics simulation (MDS) was performed for the apigenin-AKT1 complex, as this complex exhibited the highest binding affinity in the docking. Simulations were carried out using GROMACS 2020.3 with the CHARMM36 force field (Van der Spoel et al., 2005). CHARMM General Force Field (CGenFF) generated ligand topology. A simple point charge (SPC) water model in a dodecahedron box was used to solvate the protein-ligand complex and neutralized by adding three chlorine counterions. The weak Van der Waals interactions were eliminated to reduce the system’s energy using the steepest descent algorithm. Similarly, the Particle Mesh Ewald (PME) method and the linear constraint solver (LINCS) algorithm were used for constraining the electrostatic and hydrogen bond interactions. The system was equilibrated under canonical NVT (number of particles, volume, and temperature) and isobaric NPT (number of particles, pressure, and temperature) ensembles, heated to 300 K using the Berendsen thermostat with a lapse time of 0.1 ps and a pressure of 1 bar. A 100 ns simulation with a 2 fs timestep was performed. Trajectories were analyzed for root mean square deviation (RMSD), root mean square fluctuation (RMSF), hydrogen bond, radius of gyration (Rg), and solvent accessible surface area (SASA).

## 3 Results

### 3.1 Screening of Camellia sinensis active ingredients and targets

The IMPPAT 2.0 database provided a comprehensive list of phytochemicals, yielding a pool of 123 compounds with restriction applied to leaf-based phytochemicals. Filtration of compounds based on Lipinsky rule, Ghose rule, drug-likeness score (DL), and bioavailability scores (OB) (thresholds of ≥0.55 for both), resulted in a refined set of 15 compounds (Table 1).

**TABLE 1:** List of selected phytochemicals adhering to drug-like properties and absorption criteria (Lipinski’s rule of 5, Ghose rule, DLL ≥0.55, bioavailability score (OB) ≥ 0.55, and TPSA >60).

Phytochemicals	PubChem ID	Lipinski rule of 5						Lipinski rule violation	Ghosh rule	Drug likeliness	Bio availability
		MW (g/mL)	NHA	NHD	NRB	TPSA (Å)	Log P o/w				
Geranylacetone	1549778	194.31	1	0	6	17.07	4.102	yes,0	Pass	0.58	0.55
Kaempferol	5280863	286.24	6	4	1	111.13	1.965	yes,0	Pass	0.55	0.55
Apigenin	5280443	270.24	5	3	1	90.9	2.981	yes,0	Pass	0.63	0.55
Methyl 1-methoxy-1H-indole-3-carboxylate	10465540	205.21	3	0	3	40.46	2.877	yes,0	Pass	0.7	0.55
Scopoletin	5280460	192.17	4	1	1	59.67	0.859	yes,0	Pass	0.7	0.55
4-Hydroxycinnamic acid	637542	164.16	3	2	2	57.53	1.44	yes,0	Pass	0.65	0.85
Diphenyl ether	7583	170.21	1	0	2	9.23	3.739	yes,0	Pass	0.67	0.55
1,2-Dihydro-1,5,8-trimethylnaphthalene	20595	172.27	0	0	0	0	4.429	yes,1	Pass	0.56	0.55
Dihydro-beta-ionol	579336	196.33	1	1	3	20.23	3.122	yes,0	Pass	0.68	0.55
1-Isopropyl-4,7-dimethyl-1,3,4,5,6,8a-hexahydro-4a(2H)-naphthalenol	519857	222.37	1	1	1	20.23	3.606	yes,0	Pass	0.67	0.55
Cedrelanol	160799	222.37	1	1	1	20.23	4.008	yes,0	Pass	0.67	0.55
Nerolidol	8888	222.37	1	1	7	20.23	4.239	yes,0	Pass	0.63	0.55
4-(2,4,4-Trimethyl-cyclohexa-1,5-dienyl)-but-3-en-2-one	5363898	190.28	1	0	2	17.07	2.751	yes,0	Pass	0.61	0.55
Propanoic acid, 2-methyl-, 3-hydroxy-2,4,4-trimethylpentyl ester	551387	216.32	3	1	6	46.53	2.828	yes,0	Pass	0.73	0.55
1-Cyclohexen-1-ol, 2,6-dimethyl-, acetate	580935	168.12	2	0	2	26.3	2.452	yes,0	Pass	0.56	0.55

MW, molecular weight; NHA, number of hydrogen acceptor; NHD, number of hydrogen donor; NRB, number of rotatable bonds.

**FIGURE 2:**
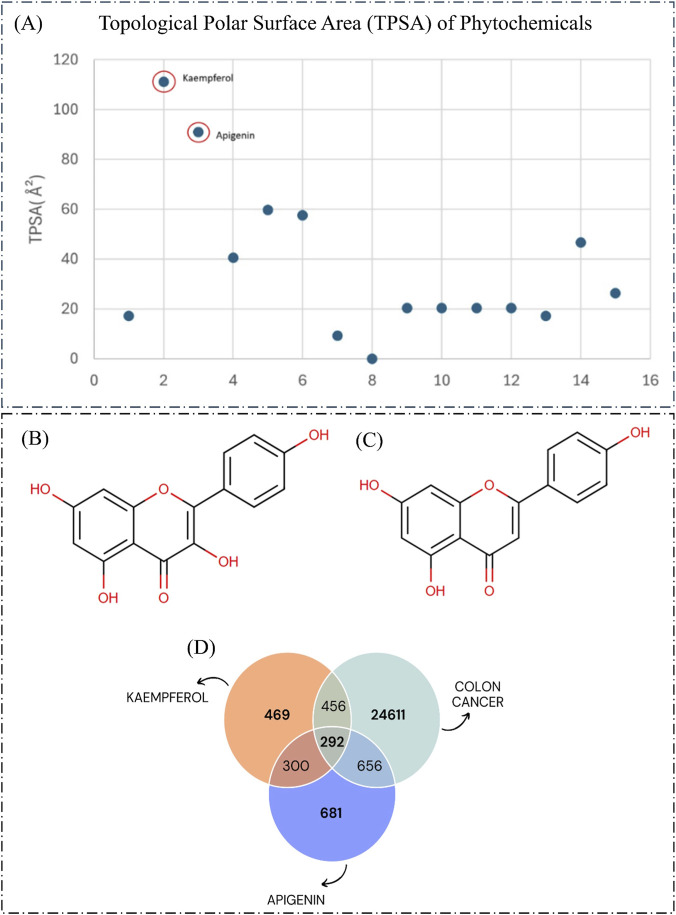
Scatter plot of phytochemicals by TPSA values- Each data point represents a phytochemical, with its position on the plot indicating its TPSA value. Kaempferol and apigenin exhibited TPSA >60 and are highlighted as potential drug delivery molecules due to their favorable physicochemical properties **(A)**. Chemical Structure of Kaempferol **(B)**, Apigenin **(C)**, Venn diagram illustrating the 292 targets of apigenin, kaempferol, and colon cancer genes **(D)**.

Further scrutiny was performed with compounds having TPSA >60 (Figure 2A). Kaempferol and apigenin are the potential drug candidates among those 15, exhibiting TPSA values of 111.13 Å^2^ and 90.9 Å^2^, respectively. Both of the candidates were the sole flavonoids identified, and the structure is depicted in Figures 2B,C. Given their adherence to all selection criteria, apigenin and kaempferol were chosen for further investigation as potential bioactive compounds from the tea plant.

Scatter plot of phytochemicals by TPSA values- Each data point represents a phytochemical, with its position on the plot indicating its TPSA value. Kaempferol and apigenin exhibited TPSA >60 and are highlighted as potential drug delivery molecules due to their favorable physicochemical properties (A). Chemical Structure of Kaempferol (B), Apigenin (C), Venn diagram illustrating the 292 targets of apigenin, kaempferol, and colon cancer genes (D).

### 3.2 Intersection and overlap analysis of gene sets

An intersection analysis identified the shared gene targets associated with apigenin, kaempferol, and colon cancer. The genes obtained from the four databases were consolidated, duplicate entries were removed, and 681 and 469 genes of apigenin and kaempferol were obtained, respectively. Further, the gene sets for apigenin and kaempferol yielded 300 common genes, suggesting a potential shared molecular target for these phytochemicals. Similarly, genes obtained from the three databases for colon cancer were integrated, and 24611 genes were obtained after manual curation of duplicates. Further analysis revealed 656 and 456 common genes of colon cancer with apigenin and kaempferol, respectively. A comprehensive intersection involving all three gene sets (apigenin, kaempferol, and colon cancer) identified 292 overlapping genes as shared targets (Figure 2D). These genes represent promising candidates for further investigation as potential therapeutic targets for colon cancer.

### 3.3 PPI network construction using STRING

The STRING database provided a comprehensive resource, resulting in a PPI network, revealing a complex network structure with a substantial number of interactions. The network comprised 273 nodes (genes) and 6607 edges (interactions) with an average node degree of 48.4. The genes, *B3GALT5*, *GPR35*, and *NEK6,* were found to be isolated nodes within the network out of 273 genes. This indicates that they do not exhibit any direct or indirect interactions with each other.

### 3.4 Network visualization and hub gene identification

**FIGURE 3:**
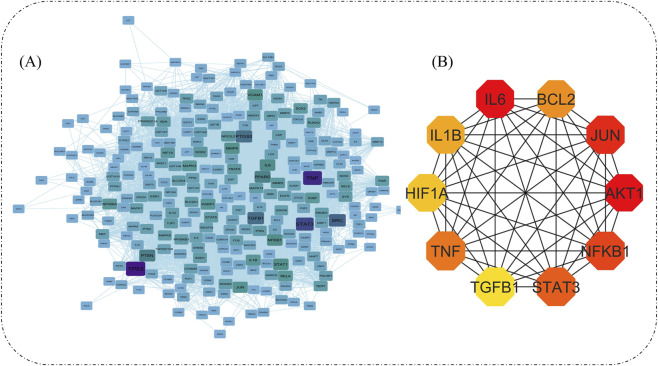
Network visualizations in Cytoscape- Compound-target protein network **(A)**. Top 10 Hub genes using CytoHubba plugin (The darker the color, the higher the score) **(B)**.

Cytoscape allows network enrichment analysis using proteins to visualize large networks. A total of 292 target proteins were obtained from the STRING online database, and visualized thorugh Cytoscape 3.10. (Figure 3A). The CytoHubba plugin, specifically designed for identifying hub genes, acquired the top 10 hub genes by applying MCC criterion, which acquires more critical proteins regarding the rank list. The 10 hub genes obtained are *AKT1*, *IL6*, *JUN*, *NFKB1*, *STAT3*, *TNF*, *BCL2*, *IL1B*, *HIF1A*, and *TGFB1* (Figure 3B). The MCODE plugin generated 9 clusters, each consisting of interconnected nodes (genes) and edges (interactions). These clusters provide valuable insights into the network’s functional organization and potential regulatory mechanisms (Table 2).

**TABLE 2:** Details of the identified clusters (score >3) from the compound target protein network.

Cluster no.	Score	Nodes	Edges	Genes involved	No. of genes
1	53.042	72	1883	*AKT1*, *APOE*, *APP*, *AR*, *ATF3*, *BCL2*, *BCL2L1*, *CASP9*, *CCL2*, *CCNB1*, *CCND1*, *CDK4*, *CDKN1A*, *CHUK*, *CRP*, *CSF2*, *CTNNB1*, *CXCL8*, *CYCS*, *EGF*, *EGFR*, *ESR1*, *ESR2*, *FGF2*, *FN1*, *FOS*, *GSK3B*, *HGF*, *HMOX1*, *HSPA5*, *ICAM1*, *IFNG*, *IGF1R*, *IL10*, *IL13*, *IL4*, *IL6*, *JUN*, *KDR*, *LEP*, *LOX*, *MAPK1*, *MAPK14*, *MAPK3*, *MAPK8*, *MET*, *MMP1*, *MMP13*, *MMP2*, *MMP3*, *MMP9*, *MPO*, *NFE2L2*, *NFKB1*, *NFKBIA*, *NOS3*, *OCLN*, *PARP1*, *PPARG*, *PTEN*, *PTGS2*, *RELA*, *RPS6KB1*, *SOCS3*, *SOX2*, *SRC*, *STAT1*, *STAT3*, *TJP1*, *TRAF6*, *VCAM1*, *XIAP*	72
2	17.659	42	362	*ABCB1*, *ABCG2*, *ARG1*, *AXL*, *CASP1*, *CASP3*, *CASP7*, *CCNA2*, *CDK1*, *CDK2*, *CDK6*, *EP300*, *ERBB2*, *GPT*, *HIF1A*, *IL1B*, *IL5*, *MAPK9*, *NANOG*, *NFKB2*, *NOS2*, *NOX4*, *NQO1*, *PGR*, *PIK3R1*, *POU5F1*, *PPARGC1A*, *PTK2*, *RUNX2*, *SELE*, *SLC2A1*, *SLC2A4*, *SOD1*, *SOD2*, *STAT6*, *SYK*, *TERT*, *TGFB1*, *TNF*, *TNFRSF10B*, *TP53*, *XDH*	42
3	13.368	20	127	*ABCC1*, *AKR1C3*, *CYP1A2*, *CYP27B1*, *CYP2C9*, *CYP2E1*, *CYP3A4*, *F2*, *GCLC*, *GSTA1*, *NR1I2*, *PPIG*, *SULT1A1*, *TSPO*, *UGT1A1*, *UGT1A10*, *UGT1A6*, *UGT1A7*, *UGT1A8*, *UGT1A9*	20
4	3.333	4	5	*ACHE*, *BACE1*, *MAPT*, *TH*	4

### 3.5 GO enrichment and KEGG pathway analysis

**FIGURE 4:**
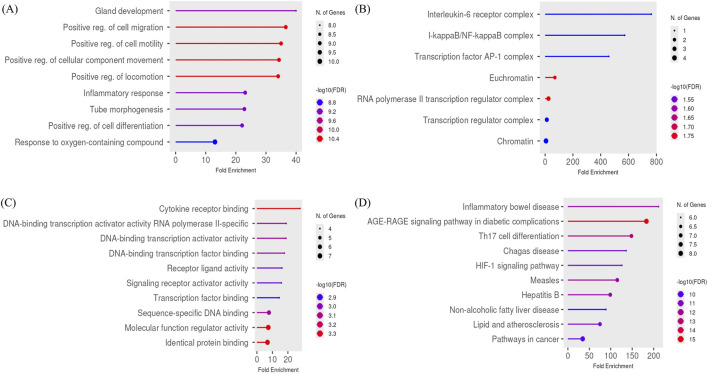
Functional enrichment pathways GO Biological process **(A)**, GO Cellular component **(B)**, GO Molecular Function **(C)**, KEGG pathway analysis **(D)**.

The KEGG pathway revealed a total of 150 pathways that are closely related to the HIF-1 signaling pathway, pathways in cancer, lipids, and atherosclerosis (Figure 4A). Our analysis revealed that the biological process contains 1000 pathways, and the key pathways are positive regulation of cell differentiation, regulation of cell migration, and tube morphogenesis (Figure 4B). Additionally, we observed enrichment in cellular component analysis related to interleukin-6 receptor complex, I-kappaB/NF-kappaB complex, and transcription factor AP-1 complex, with 23 pathways (Figure 4C). Furthermore, in the molecular function pathway enrichment, 97 pathways are associated with key pathways such as DNA binding transcription activator activity, sequence-specific DNA binding, ubiquitin protein ligase binding, and cytokine receptor binding (Figure 4D). This comprehensive analysis provides valuable insights into the potential mechanisms of action of the two drugs and their impact on key cellular processes.

### 3.6 Mutation frequency analysis

**FIGURE 5:**
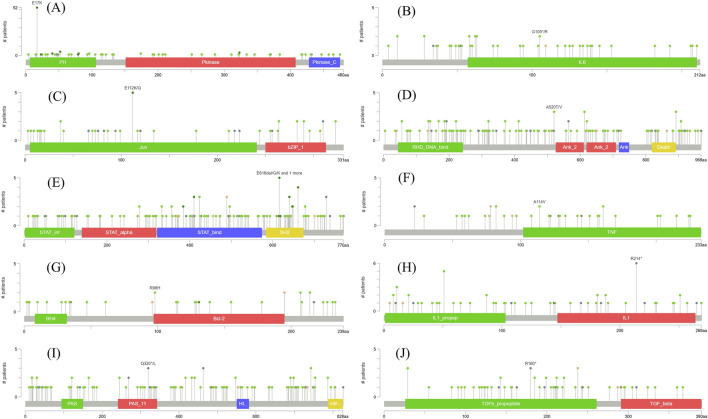
Genomic mutation map of hub genes- *AKT1* **(A)**, *IL6* **(B)**, *JUN* **(C)**, *NFKB1* **(D)**, *STAT3* **(E)**, *TNF* **(F)** *BCL2* **(G)**, *IL1B* **(H)**, *HIF1A* **(I)**, *TGFB1* **(J)**.

The mutation frequency maps were obtained from cBioPortal (Figure 5). *AKT1*, *IL6*, *JUN*, *NFKB1*, *STAT3*, *TNF*, *BCL2*, *IL1B*, *HIF1A*, and *TGFB1* exhibited mutation frequencies of 1.85%, 1.18%, 0.67%, 2.19%, 1.35%, 0.17%, 0.51%, 1.01%, 2.19%, and 1.18%, respectively. The mutation frequency, deep deletion, and amplifications was added up to determine the gene modification rates. These rates of the hub genes were found to be 2.19%, 1.68%, 0.67%, 2.53%, 2.02%, 0.84%, 2.36%, 1.01%, 2.19%, and 1.35%. From the analysis, it was observed that *AKT1*, *NFKB1*, and *HIF1A* have the highest mutation frequencies of 1.85%, 2.19%, and 2.19%, respectively. These three genes were given priority for further experiments because of their substantial role in COAD.

### 3.7 Molecular docking analysis

**FIGURE 6:**
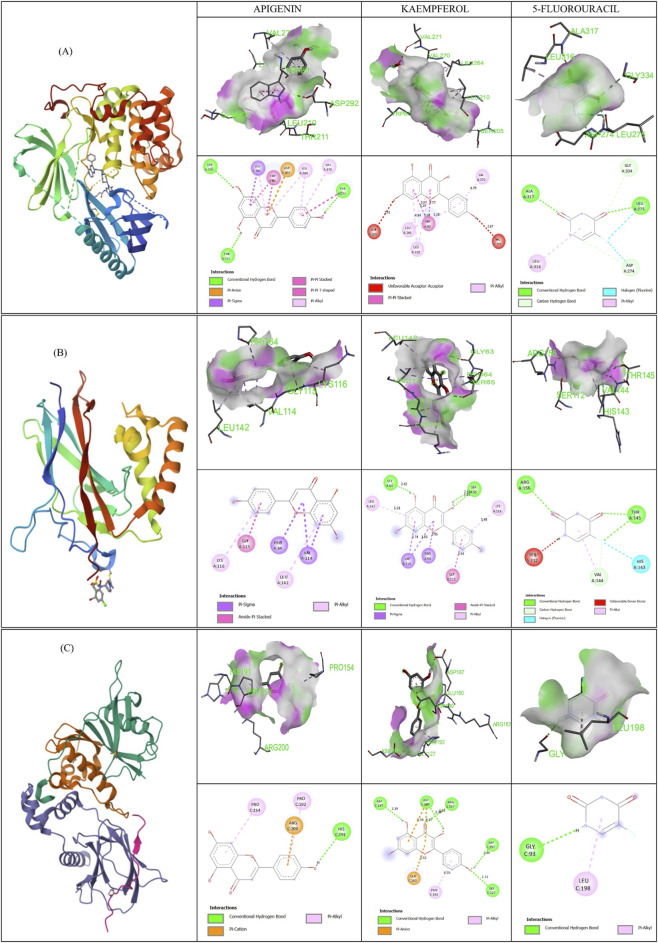
2D and 3D interaction and binding models of the three selected genes depicting the hydrogen bonds and the attached amino acids, visualized using Biovia- *AKT1* - 6HHF **(A)**, *NFKB1* - 8TQD **(B)**, *HIFA* - 1LQB **(C)**, with apigenin, kaempferol, and 5-fluorouracil.

Based on mutation frequency, three hub genes - *AKT1*, *NFKB1*, and *HIF1A* were chosen for docking to identify the binding affinity between the hub genes and the ligands. The three hub genes were docked with two active compounds, apigenin and kaempferol. The 3D and 2D binding interactions between the proteins and ligands are shown in Figure 6. Table 3 provides detailed binding affinity values and information on the amino acid residues involved in the interactions.

**TABLE 3:** Binding energies of target genes and ligands based on molecular docking analysis.

Gene	PDB number	Compound	Amino acid count	Names of amino acids	Binding energy
*AKT1*	6HHF	Apigenin	8	SER205, THR211, LEU210, TRP80, ASP292, LEU264, VAL270, TYR272	−9.4
		Kaempferol	6	SER205, LEU264, LEU210, TRP80, VAL270, VAL271	−9.2
		5 fluorouracil	5	ALA317, LEU316, GLY334, LEU275, ASP274	−6
*NFKB1*	8TQD	Apigenin	5	VAL114, LEU142, PRO64, GLY115, LYS116	−6.8
		Kaempferol	7	SER65, VAL114, LEU142, PRO64, GLY115, LYS116, GLY63	−7.2
		5 fluorouracil	5	ARG156, SER112, VAL144, HIS143, THR145	−4.4
*HIF1A*	1LQB	Apigenin	4	PRO154, ARG200, PRO192, HIS191	−7.2
		Kaempferol	7	ASP187, ASP190, GLU160, ARG167, PRO192, ASP197, GLY127	−6.6
		5 fluorouracil	2	GLY93, LEU198	−4.3

The predicted binding energies for apigenin with genes *AKT1*, *NFKB1*, and *HIF1A* were −9.4, −6.8, and −7.2 kcal/mol, respectively. Kaempferol showed binding energies of −9.2, −7.2, and −6.6 kcal/mol for *AKT1*, *NFKB1*, and *HIF1A* genes, respectively. The standard chemotherapy drug 5-Fluorouracil showed binding energies of −6.0, −4.4, and −4.3 kcal/mol with *AKT1*, *NFKB1*, and *HIF1A* genes.

### 3.8 Analysis of binding free energy

The Uni-GBSA–based binding free-energy analysis further supported the docking results. Apigenin showed predicted binding free energies of −28.54, −18.26, and −20.41 kcal/mol with AKT1, NFKB1, and HIF1A, respectively. Kaempferol exhibited binding free energies of −27.42, −20.36, and −17.41 kcal/mol for the same target proteins. The standard drug 5-Fluorouracil (5-FU) demonstrated comparatively weaker binding, with binding free-energy values of −15.85, −10.27, and −10.32 kcal/mol for AKT1, NFKB1, and HIF1A, respectively. Overall, both flavonoids displayed more favorable MM/GBSA binding energies than 5-FU, indicating stronger potential interactions with these colon–cancer–associated targets.

### 3.9 Molecular dynamics simulation

**FIGURE 7:**
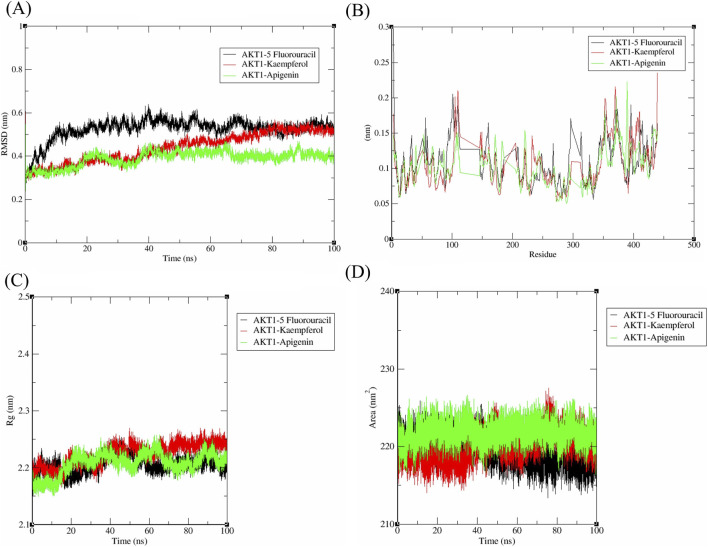
MD simulation analysis of *AKT1* in complex 5-Fluorouracil, Kaempferol, and Apigenin- RMSD **(A)**, RMSF **(B)**, Radius of gyration **(C)**, SASA **(D)**.

The dynamic behaviour of *AKT1* complexes was extensively analyzed through MD simulations. The results are shown in Figure 7. The *AKT1*-5-Fluorouracil complex is represented by a black trendline, while the *AKT1*-Kaempferol complex and the *AKT1*-Apigenin complex are depicted by red and green trendlines, respectively. The RMSD trajectory of *AKT1*- 5-Fluorouracil complex exhibits a sharp increase from 0.4 nm to 0.5 nm, approximately, resulting in 0.537 nm at 100th ns. In contrast, *AKT1*-Kaempferol complex shows an RMSD value of 0.521 nm at 100th ns, achieving stabilization at 80 ns despite minor deviations across the time intervals. *AKT1*-Apigenin complex demonstrates the lowest RMSD (0.409 nm) among the investigated complexes with minimal fluctuations before reaching stability around 70 ns, indicating the highest structural stability within the binding site of *AKT1* (Figure 7A). The key binding site residues of *AKT1* include Leu156, Val164, Ala177, Met227, Tyr229, Ala230, Phe236, Phe237, Met281, Tyr437, Phe438, and Phe442 characteristics were explored during simulation. *AKT1*-5-Fluorouracil and *AKT1*-Kaempferol complexes showed ∼0.089 nm RMSF, each, indicating more flexibility and weaker binding within the residues. But, *AKT1*-Apigenin shows a lower RMSF of ∼0.083 nm, showing higher stability and minimal fluctuations. These complexes form interactions with Thr211 and Asp292, which is crucial for AKT1 function, showing stability (Figure 7B), that align well with RMSD and docking results.

In the radius of gyration (Rg) trajectory, all three 3 complexes exhibited a similar trend with minor deviations (Figure 7C). *AKT1*-5-Fluorouracil complex showed a consistent compactness throughout the simulation, which resulted in Rg value of 2.196 nm *AKT1*-Kaempferol complex exhibited a slightly higher Rg of 2.237 nm, indicating a relatively expanded conformation, while *AKT1*-Apigenin complex maintained a stable trend with fluctuations similar to the other two compounds and a lesser Rg of 2.195 nm *AKT1*-5-Fluorouracil complex exhibits the lowest SASA values (219.298 nm^2^), indicating reduced solvent exposure and a more compact conformation. In contrast, *AKT1*-Kaempferol and *AKT1*-Apigenin complexes displayed higher SASA values - 218.618 nm^2^ and 219.938 nm^2^, respectively, with *AKT1*-Apigenin complex showing the highest solvent accessibility, suggesting a better structural flexibility and an enhanced potential for interactions with the solvent environment (Figure 7D).

## 4 Discussion

To date, chemotherapy, radiation, and surgery are the treatment options for colon cancer, but are associated with adverse effects, compromising patients’ lives, with a driving interest in natural compounds with anticancer potential and lower toxicity profiles (Chaudhary et al., 2023). In this study, we investigated molecular targets of bioactive compounds from the tea plant (*C. sinensis*), using network pharmacology and bioinformatics tools.

Based on their higher TPSA values (optimal range: 60–140 Å^2^), apigenin and kaempferol were selected as phytochemicals (Prasanna and Doerksen, 2009). Apigenin is known for inhibiting cell growth and metastasis through modulating PI3K/AKT1, MAPK/ERK, and JAK/STAT pathways. Apigenin were given in controlled quantities in combination for colorectal carcinoma patients to prevent relapse (Yan et al., 2017). Similarly, kaempferol induced apoptosis, cell cycle arrest, and inhibited angiogenesis and metastasis, primarily through *VEGF* targeting and epithelial-mesenchymal transition (EMT) proteins (Kashyap et al., 2017).

Target genes related to compounds and colon cancer were screened, and common targets that provide insights into drug efficacy were identified (Neary et al., 2021). 273 genes with 6607 nodes, showing high gene interaction, were screened through STRING (Szklarczyk et al., 2019). Among these, *B3GALT5*, *GPR35*, and *NEK6* showed no interactions within the network. The plugins of Cytoscape, namely, MCODE and CytoHubba, identified key clusters and hub genes, respectively (Nangraj et al., 2020; Lu et al., 2023). MCC gives priority to proteins that participate in the fully connected subnetworks, in identifying the top 10 hub genes (Li and Xu, 2019).

Functional enrichment analysis showed hub genes associated with pathways regulating interleukin-6 receptor signaling, NF-kappaB complex, transcription factor AP-1 complex, positive regulation of cell migration, and inflammatory response. These results suggest high compatibility with the GO terms linking the specific gene functions (Yang et al., 2014). KEGG pathway analysis further illustrated molecular interactions and biochemical networks (Kanehisa et al., 2012), associating target genes with pathways such as the HIF-1 signaling pathway, Th17 cell differentiation, and cancer-related pathways (Yang et al., 2014), reinforcing their therapeutic relevance. Mutation frequency examination has been shown to correlate with cancer detection and prognosis (Loeb et al., 2003). Identification and targeting of highly mutated genes in the tumor cells can enhance the efficacy of drugs to push the cell beyond its critical point of viability, leading to the tumor cell death (Yu et al., 2004). Using cBioportal, it was observed that *AKT1*, *NFKB1*, and *HIF1A* exhibited the highest mutation frequencies, suggesting these as critical targets in cancer therapy.

Akt regulates multiple cellular processes, including metabolism, proliferation, and growth, and functions as a central mediator of cell survival by suppressing apoptotic signaling; accordingly, it is said to be “survival kinase.” The phosphoinositide 3-kinase/v-akt murine thymoma viral oncogene/mammalian target of the rapamycin (PI3K/AKT/mTOR) pathway is dysregulated in colorectal carcinoma through multiple mechanisms, including activating missense mutations in *PIK3CA* and *AKT1*, as well as loss of PTEN expression (Hechtman et al., 2015). NF-κB1 and HIF-1A are recognized therapeutic targets in metastatic colorectal cancer. Aberrant activation of NF-κB signaling promotes tumorigenesis by enhancing cell proliferation and angiogenesis, while suppressing apoptosis. HIF-1α plays a critical role in cancer progression and is activated in most cancers. Although HIF-1α expression is minimal in most normal tissues, both HIF-1α and HIF-2α are markedly upregulated in tumor tissues (Demirkiran et al., 2023).

Molecular docking is used for examining how drugs interact with the target genes (Vodenkova et al., 2020). Using this technique, we evaluated the binding affinity of drug molecules with the target genes against 5-fluorouracil (standard). Both apigenin and kaempferol show the highest binding affinity towards *AKT1*, highlighting that combinational therapy will be an advantage over monotherapy, improving the therapeutic success (Plana et al., 2022). Simulation shows the behavior of proteins and nucleic acids at the atomic level (Badar et al., 2022). From the simulation studies, the apigenin-AKT1 complex showed better stability than kaempferol-AKT1.

This study is primarily based on computational biology, integrating network pharmacology, molecular docking, and molecular dynamics simulations. Though the approaches are valuable for target identification and hypothesis generation, they do not provide direct experimental validation of compound efficacy or pathway modulation in colon cancer models. Molecular dynamics simulations were performed for the apigenin–AKT1 complex only, based on docking performance, and were not extended to all predicted targets or ligands. Therefore, *in vitro* and *in vivo* experimental studies are required to validate the predicted interactions, assess biological activity, and confirm therapeutic relevance in colon cancer.

## 5 Conclusion

Integrating network pharmacology and bioinformatic analyses showed the potential of phytochemicals in colon cancer. The identification of ten key hub genes was majorly involved in proliferation, apoptosis, inflammation, and angiogenesis. Among these, the highly mutated genes such as *AKT1*, *NFKB1*, and *HIF1A* were prioritized as the targets. Molecular docking revealed strong binding affinities of apigenin and kaempferol to *AKT1* compared with 5-Fluorouracil, with molecular dynamics confirming apigenin as the most stable and effective binder. The study shows the potential of Apigenin and kaempferol as phytochemical-based therapeutics against colon cancer, and a thorough experimental validation is essential for clinical translation.

## Data Availability

The original contributions presented in the study are included in the article, further inquiries can be directed to the corresponding author.
